# Immunotherapy for Infarcts: *In Vivo* Postinfarction Macrophage Modulation Using Intramyocardial Microparticle Delivery of *Map4k4* Small Interfering RNA

**DOI:** 10.1089/biores.2020.0037

**Published:** 2020-12-02

**Authors:** Jun Luo, Matthew S. Weaver, Timothy P. Fitzgibbons, Myriam Aouadi, Michael P. Czech, Margaret D. Allen

**Affiliations:** ^1^ Matrix Biology Program, Benaroya Research Institute at Virginia Mason, Seattle, Washington, USA.; ^2^ Cardiovascular Medicine, Department of Medicine, University of Massachusetts Medical School, Worcester, Massachusetts, USA.; ^3^ Integrated Cardio Metabolic Center, Department of Medicine, Karolinska Institutet, Huddinge, Sweden.; ^4^ Program in Molecular Medicine, University of Massachusetts Medical School, Worcester, Massachusetts, USA.; ^5^ Division of Cardiothoracic Surgery, Department of Surgery, University of Washington, Seattle, Washington, USA.

**Keywords:** macrophage, microparticle, siRNA, myocardial infarction, Map4k4, intramyocardial

## Abstract

The myeloid cells infiltrating the heart early after acute myocardial infarction elaborate a secretome that largely orchestrates subsequent ventricular wall repair. Regulating this innate immune response could be a means to improve infarct healing. To pilot this concept, we utilized (β1,3-d-) glucan-encapsulated small interfering RNA (siRNA)-containing particles (GeRPs), targeting mononuclear phagocytes, delivered to mice as a one-time intramyocardial injection immediately after acute infarction. Findings demonstrated that cardiac macrophages phagocytosed GeRPs *in vivo* and had little systemic dissemination, thus providing a means to deliver local therapeutics. Acute infarcts were then injected *in vivo* with phosphate-buffered saline (PBS; vehicle) or GeRPs loaded with siRNA to *Map4k4*, and excised hearts were examined at 3 and 7 days by quantitative polymerase chain reaction, flow cytometry, and histology. Compared with infarcted PBS-treated hearts, hearts with intrainfarct injections of siRNA-loaded GeRPs exhibited 69–89% reductions in transcripts for Map4k4 (mitogen-activated protein kinase kinase kinase kinase 4), interleukin (IL)-1β, and tumor necrosis factor α at 3 days. Expression of other factors relevant to matrix remodeling—monocyte chemoattractant protein-1 (MCP-1), matrix metalloproteinases, hyaluronan synthases, matricellular proteins, and profibrotic factors transforming growth factor beta (TGF-β), and connective tissue growth factor (CTGF)—were also decreased. Most effects peaked at 3 days, but, in some instances (Map4k4, IL-1β, TGF-β, CTGF, versican, and periostin), suppression persisted to 7 days. Thus, direct intramyocardial GeRP injection could serve as a novel and clinically translatable platform for *in vivo* RNA delivery to intracardiac macrophages for local and selective immunomodulation of the infarct microenvironment.

## Introduction

Macrophages and monocytes (M/Ms) accumulate rapidly in the heart after acute myocardial infarction (AMI) and largely orchestrate postinfarct ventricular remodeling^1,2^ ; yet, current therapies have not addressed this innate immune response. The first wave of infiltrating M/Ms elaborate proinflammatory cytokines that trigger degradation of extracellular matrix and initiate profibrotic processes which, unchecked, contribute to ventricular wall dilatation, interstitial fibrosis, and heart failure.^3^ Thus, modulating the behavior of this early intracardiac myeloid population could provide an opportunity to influence and optimize postinfarct myocardial recovery.

These pilot experiments explored whether glucan-encapsulated small interfering RNA (siRNA)-containing particles (GeRPs), developed by Czech and colleagues,^4–7^ could be utilized for *in situ* modification of M/Ms within the heart. The β1,3-d-glucan shells of these 2–4 μm-sized particles bind dectin-1 receptors on phagocytes,^8^ selectively targeting siRNA uptake to M/Ms.^5^ Furthermore, RNA interference (RNAi) screening previously identified the silencing of *Map4k4* (mitogen-activated protein kinase kinase kinase kinase 4) as being particularly effective at downregulating inflammatory cytokine release in peritoneal macrophages.^4^ An 8-day course of orally delivered GeRPs resulted in efficient particle ingestion by macrophages in gut-associated lymphoid tissues, which then trafficked to systemic lymphatic organs. *Map4k4* knockdown inhibited interleukin (IL)-1β and tumor necrosis factor (TNF)-α production in peritoneal macrophages, significantly reduced systemic inflammatory signaling, and protected mice from LPS-induced lethality.^4^ Courses of intraperitoneally and intravenously delivered GeRPs, using other siRNAs, have effectively silenced inflammatory genes in adipose macrophages and Kupffer cells, ameliorating insulin resistance.^7,9^

However, for the AMI setting, the objective was instead to selectively target M/Ms within the heart, not systemically, to achieve a local, time-limited dampening of early inflammatory responses within the infarct. We hypothesized that injecting a single dose of siRNA-loaded GeRPs directly into the infarct site would create a repository of intramyocardial particles encountered only by the early M/Ms infiltrating the heart, and cardiac-resident macrophages. Specifically, we examined whether intracardiac M/Ms would phagocytose GeRPs deposited in acute infarcts; whether macrophages with ingested particles would remain in the heart or return to the circulation; and whether a single dose of *Map4k4*-directed siRNA could modify M/M secretory profiles across the entire myocardium, recognizing that only a fraction of the intracardiac M/M population would take up particles.

## Methods

### Animals

Animal protocols were approved by the Institutional Animal Care and Use Committee under NIH guidelines. C57BL/6J mice, age 7–9 weeks, 20–26 g, were purchased from Jackson Laboratory.

### Particles

GeRPs were prepared as described previously.^4–7^ In brief, hollow glucan shells, 2–4 μm diameter, produced from *Saccharomyces cerevisiae* cell walls, were labeled with 5-DTAF [5-(4, 6-dichlorotriazinyl) aminofluorescein; ThermoFisher]. For siRNA loading, 10 μL of 1 mM siRNA were combined with 85 μL of sodium acetate (30 mM), then added to 94 μL of 5 mM Endoporter (Gene Tools), and incubated at room temperature for 15 min. Ten milligrams of glucan shells were added, incubated for 1 h at room temperature, and then diluted in phosphate-buffered saline (PBS) to 1.5 mL. Particles were stored at −80°C and sonicated before use. Heart injectates of empty particles (no siRNA) utilized a dose of 2.7 × 10^8^ β-glucan shells/heart. Injectates of siRNA-loaded particles consisted of 5 × 10^7^ GeRPs in 10 μL PBS, containing 50 pmol *Map4k4* siRNA, calculated to provide 10 GeRPs/macrophage, based on 4 × 10^6^ F4/80^+^ macrophages/heart measured on day 2 postinfarction. The *Map4k4* siRNA sequence utilized was 5′-GACCAACUCUGGCUUGUUAUU-3′, accession number NM_008696.2.^4^

### Myocardial infarction and intramyocardial injections

Under isoflurane anesthesia with mechanical ventilation, AMIs were created through left thoracotomies by left anterior descending coronary ligation (without reperfusion). Five minutes later, a single 10 μL injection of PBS, empty GeRPs (no siRNA), or siRNA-loaded GeRPs was made directly into the infarct center using a 30G needle. At terminal surgery, mice were re-anesthetized with isoflurane for tissue collection.

### Flow cytometry

After heparinization, peripheral blood, spleen, heart, and femurs were collected. Hearts were perfused with 2 mL 4°C St. Thomas' cardioplegia solution, atria discarded, and the entire ventricle minced, then suspended in 8–10 mL lysis buffer (10 mM HEPES, 30 mM taurine, and 0.2 mg/mL Liberase TH protease cocktail [Roche] in HBSS [1 × ]). The suspension was filtered (40 μm strainer) into digestion-stopping buffer (10 mM HEPES, 30 mM taurine, diluted 1:10 with heat-inactivated fetal bovine serum). Spleen tissue and bone marrow were similarly filtered. Heart, spleen, and marrow-isolated cells were pelleted by centrifugation (5 min, 1000 × *g*, 4°C), incubated in erythrocyte lysis buffer (BioLegend), re-pelleted and washed in 1 mL cell staining buffer (BioLegend), centrifuged and resuspended in 50 μL of staining buffer containing rat anti-mouse antibodies against CD11b (M1/70, PerCP/Cy5.5-conjugated), F4/80 (BM8, PE-conjugated), and Ly-6C (HK1.4, PE-Cy7-conjugated; all BioLegend). Twenty thousand events were collected/sample on the flow cytometer (LSRII; BD Biosciences), and analyzed with FlowJo v10.0.7 (Tree Star). Macrophages were defined as CD11b^high^/F4/80^high^ with internalized GeRP particles identified by fluorescein labels. Ly6C expression was categorized as high, intermediate, or low, reporting CD11b^high^/F4/80^high^ macrophages with high and low Ly6C expression. Macrophages with intermediate intensity Ly6C labeling, often comprising a majority of cells, were not included in this polarized analysis.

### Quantitative polymerase chain reaction

Mouse hearts were perfused with 2 mL 4% paraformaldehyde (PFA) at 4°C, excised, atria discarded, and the right and left ventricles (infarcted plus noninfarcted myocardium), placed in RNAlater (Qiagen), and stored at −80°C. After thawing, the RNeasy Fibrous Tissue Mini Kit (Qiagen) was utilized, placing tissue in 300 mL RLT buffer (Qiagen), then homogenizing for 45–60 sec (Omni TH; OMNI International). RNA concentration was determined on a NanoDrop spectrophotometer (ThermoFisher) and 1 μg RNA used as template in a 50 μL reverse transcription polymerase chain reaction (PCR) using a High Capacity complementary DNA (cDNA) Reverse Transcription Kit (Applied Biosytems). Subsequently, 1 mL of each cDNA sample was used as a template in quantitative PCR with primers for SYBR Green labeling (for murine genes encoding Map4k4 [*Map4k4*], TNF-α [*Tnf*], IL-1β [*Il1b*], monocyte chemoattractant protein-1 [MCP-1, *Ccl2*], matrix metalloproteinase [MMP]-1 [*Mmp1a*], MMP-2 [*Mmp2*], MMP-9 [*Mmp9*], MMP-12 [*Mmp12*], insulin-like growth factor-1 [IGF-1, *Igf1*], Has-1 [*Has1*], Has-2 [*Has2*], HYAL-1 [*Hyal1*], CD44 [*Cd44*], connective tissue growth factor [CTGF, *Ctgf*], fibronectin [*Fn1*], collagen III [*Col3a1*], SPARC [*Sparc*], tenascin-C [*Tnc*], and periostin [*Postn*]; Applied Biosystems) or TaqMan (versican [*Vcan*], transforming growth factor beta [TGF-β, *Tgfb1*], collagen I [*Col1a1*], IL-10 [*Il10*], and 18S ribosomal RNA [rRNA, *Rn18s*]; Applied Biosystems). Reactions were run for 45 cycles and results read on ABI 7900HT real-time equipment (Applied Biosystems). Delta Ct (ΔCt) was calculated as the fold difference in cycles (Ct) for the target genes referenced to the 18S rRNA gene. Relative messenger RNA (mRNA) levels were calculated by the 2^−ΔΔCt^ method^10^ to express the fold increase over RNA values in untreated (noninfarcted) murine hearts.

### Histology

Hearts were perfused *in situ* with 2mL 4% PFA at 4°C, excised, and placed in 4% PFA at 4°C for 24 h, then washed in 70% EtOH, sliced transversely, paraffin embedded, and sectioned at 5 μm intervals. Immunohistochemistry utilized antibodies to F4/80 (Cl:A3–1; Abcam) for murine macrophages; and TNF-α (52B83; Santa Cruz); cell nuclei were counterstained with Hoeschst dye (ThermoFisher). Fluorescein labels identified GeRPs. Hyaluronan (HA) was visualized by biotin-conjugated HA-binding protein followed by streptavidin-Texas Red containing 1% bovine serum albumin.^11^

### Statistical analysis

Mean values were compared between treatment groups using two-tailed Student's *t* tests, or one-way analysis of variance with Tukey's *post hoc* test for multiple comparisons. Significance was determined at *p* < 0.05 and variance presented as ± standard error of the mean.

## Results

### Acute infarction increases GeRP uptake by cardiac macrophages

To examine the effects of AMI on GeRP phagocytosis, a single dose of 2.7 × 10^8^ fluorescein-labeled empty GeRP particles (without siRNA) was injected into infarct centers 5 min after infarct creation (*n* = 4) or into noninfarcted left ventricular walls (*n* = 2). At 7 days, GeRP uptake across all cardiac macrophages was assessed by flow cytometry. The prevalence of F4/80^high^ macrophages was 60% higher in infarcted versus noninfarcted hearts (10% ± 2% vs. 6% ± 1% of all live cells, including cardiomyocytes). In infarcted hearts, 53% ± 6% of F4/80^+^ macrophages coexpressed fluorescein labels, indicating GeRP phagocytosis, whereas in noninfarcted hearts, only 10% ± 3% were fluorescein labeled (*p* < 0.01; Supplementary Figs. S1 and S2).

### Biodistribution of macrophages with internalized GeRPs

**FIG. 1.**
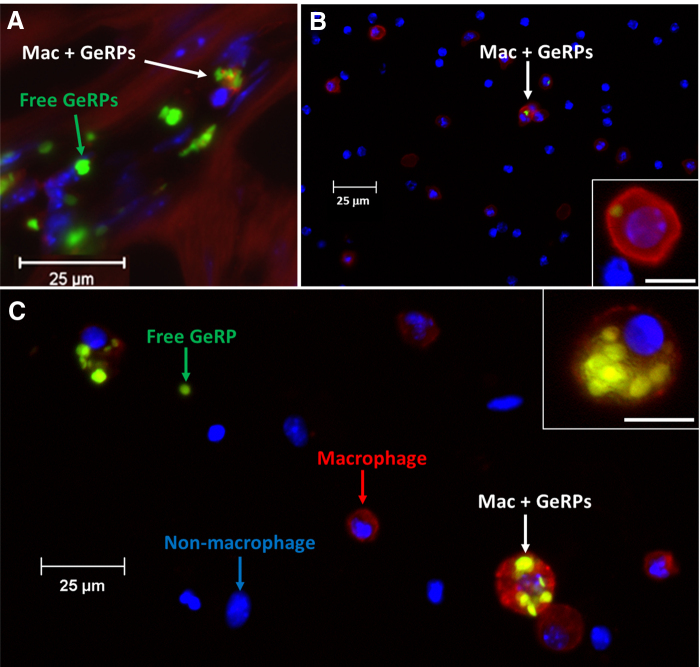
GeRP phagocytosis by macrophages. **(A)** Representative histologic cross-section of infarcted myocardium at 7 days after infarction and GeRP injection, demonstrating macrophages with internalized GeRP particles, as well as free GeRP particles, surrounded by cardiomyocytes. Immunostaining with antibody to F4/80 identifies macrophages (red), whereas the fluorescein label on GeRPs appears green. Internalized GeRPs were not observed in somatic cells (i.e., cardiomyocytes). **(B, C)** Representative cell isolates from spleen **(B)** and myocardium **(C)** at 7 days after infarction, illustrating free and internalized GeRPs (yellow-green) and macrophages (F4/80^+^, red staining). Insets show examples of F4/80^+^ cells with internalized GeRPs: **(C)** those from infarcted myocardial tissue have ingested multiple GeRPs per cell in contrast to the rare, single GeRPs found in splenic macrophages **(B)**. Scale bars demonstrate the relative paucity of GeRP^+^ macrophages among splenic **(B)** versus myocardial **(C)** cells. GERPs, glucan-encapsulated small interfering RNA-containing particles.

By 1 week after AMI and intramyocardial GeRP injection, low percentages of F4/80^+^ cells with internalized GeRPs were found in peripheral blood (5% of all live cells), bone marrow (3%), and spleen (2%). In animals without infarction, these extracardiac sites had even lower frequencies of GeRP-containing macrophages at 3%, 2%, and 0.4%, respectively. Percentages of F4/80^+^ macrophages with internalized GeRPs were significantly lower in extracardiac sites than in either infarcted hearts (*p* < 0.0001) or noninfarcted hearts (*p* < 0.03; Supplementary Fig. S2). On histology, macrophages within infarcted hearts often contained multiple GeRPs per cell (Fig. 1), whereas at extracardiac sites, the few macrophages with internalized GeRPs usually phagocytosed only single particles (Fig. 1).

### Uptake of siRNA-loaded GeRPs by intramyocardial M/Ms in infarcted hearts

To pilot siRNA delivery to intracardiac macrophages, single doses of 5 × 10^7^ GeRP particles, containing 50 pg of *Map4k4* siRNA (*n* = 3/group), or PBS (*n* = 6/group), were injected into infarct centers 5 min after AMI. At 3 and 7 days after infarction and injection, excised hearts were analyzed by FACS to gauge GeRP ingestion among CD11b^+^-gated F4/80^+^ (macrophage) and F4/80^−^ (monocyte) populations. At 3 days, 21% ± 5% of cardiac CD11b^+^/F4/80^+^ macrophages coexpressed fluorescein labels, indicating GeRP phagocytosis, which by 7 days, had dropped to 13% ± 3%. Among CD11b^+^/F4/80^−^ intracardiac monocytes, a similar percentage, 22% ± 7%, contained GeRPs by 3 days, which also diminished to 10% ± 4% by 7 days.

### Effects of *Map4k4*-siRNA-loaded GeRPs on macrophage phenotype

**FIG. 2.**
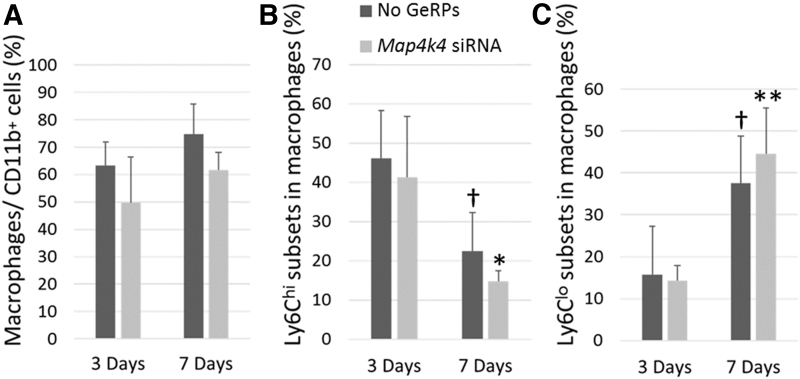
Prevalence of macrophages and Ly6C-expressing subsets following infarction and intrainfarct GeRP delivery of *Map4k4* siRNA. **(A)** Percentage of F4/80^hi^ cells, denoting macrophages, among all CD11b^hi^ leukocytes on FACS analysis of digested whole hearts at 3 and 7 days after infarction and intramyocardial injection of GeRPs containing *Map4k4* siRNA (light gray) or vehicle (dark gray). **(B, C)** Percentages of Ly6C^hi^- and Ly6C^lo^-expressing cells among the subset of CD11b^hi^/F4/80^hi^ macrophages at 3 and 7 days in the same hearts. Data are presented as mean values ± SEM. *p < 0.05 versus GeRP-treated infarcted hearts at 3 days; **p = 0.01 versus GeRP-treated infarcted hearts at 3 days; ^†^*p* < 0.01 versus PBS-treated infarcted hearts at 3 days. PBS, phosphate-buffered saline; SEM, standard error of the mean; siRNA, small interfering RNA.

Ly6c expression was examined to explore whether intramyocardial GeRP delivery would alter phenotypes across the entire cardiac macrophage population. The proportion of F4/80^+^ macrophages among CD11b^+^ leukocytes was reduced by 20% at both 3 and 7 days in GeRP-treated infarcted hearts compared with infarcted controls (*p* = 0.1; Fig. 2). On subset analysis, a statistically significant switch from predominantly Ly6c^hi^-expressing macrophages, typically considered a proinflammatory phenotype,^12^ to Ly6c^lo^ expression, classically a reparative characteristic,^3,13^ was evident in PBS-treated infarcted hearts between the 3- and 7-day time points (*p* < 0.01, Fig. 2). Parallel reductions in Ly6c^hi^-expressing phenotypes (*p* < 0.05) and elevations in Ly6c^lo^-expressing ones (*p* = 0.01) were seen in GeRP-treated hearts (Fig. 2). At both 3 and 7 days, proportions of M1/M2 macrophage subpopulations in GeRP-treated hearts were not significantly different from those in PBS-treated hearts.

### *Map4k4* silencing after intramyocardial injection of *Map4k4*-siRNA-loaded GeRPs

**FIG. 3.**
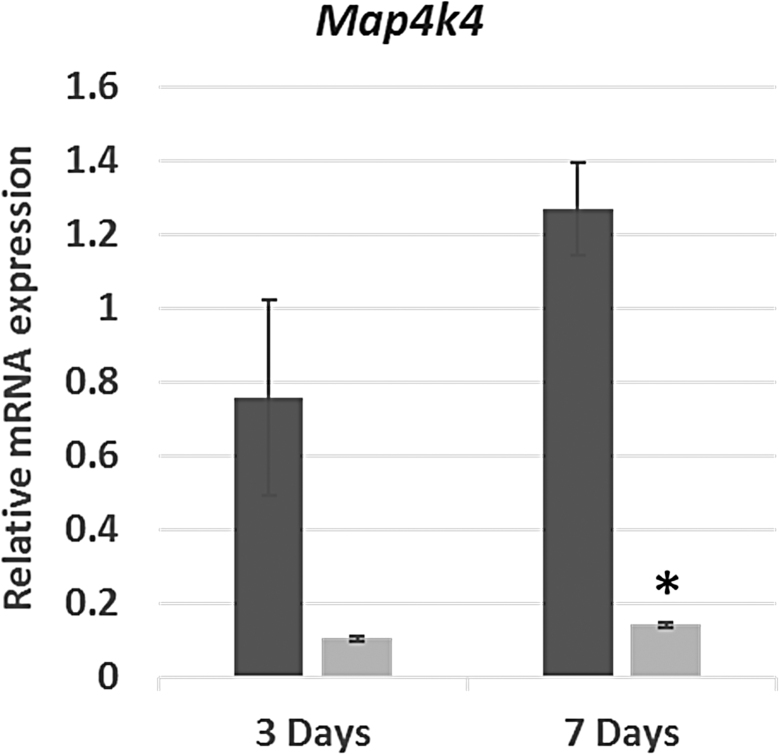
Relative *Map4k4* gene expression after acute myocardial infarction and GeRP delivery of *Map4k4* siRNA. Abundance of *Map4k4* RNA in acutely infarcted hearts injected immediately after infarction with a single dose of PBS (vehicle, dark gray) or GeRPs containing *Map4k4* siRNA (light gray). Data are presented as mean values (± SEM) relative to expression in hearts without infarction or surgery as normal controls. *p < 0.001 versus PBS-treated infarcted hearts at 7 days. Map4k4, mitogen-activated protein kinase kinase kinase kinase 4.

Total cardiac RNA from both ventricles (infarcted plus noninfarcted myocardium) was analyzed to determine whether infarct-injected *Map4k4-*siRNA-containing GeRPs would impact *Map4k4* gene expression across the heart. Hearts injected with *Map4k4-*siRNA-loaded GeRPs (*n* = 3/group) or PBS (*n* = 5–6/group) were procured at 3 or 7 days after infarction; normal hearts without infarction or surgery served as reference controls (*n* = 8). *Map4k4* transcripts in hearts treated with *Map4k4*-siRNA-GeRP injections were reduced by 86% at 3 days and 89% at 7 days (*p* < 0.001) compared with PBS-treated infarcted hearts (Fig. 3).

### Effects of *Map4k4*-siRNA-loaded GeRP delivery on inflammatory mediators

The same hearts were analyzed for expression of a panel of factors with known relevance for infarct healing. Again, transcripts were evaluated over the entire heart, not just the infarct area, as the end-point most likely to influence infarct remodeling.

**FIG. 4.**
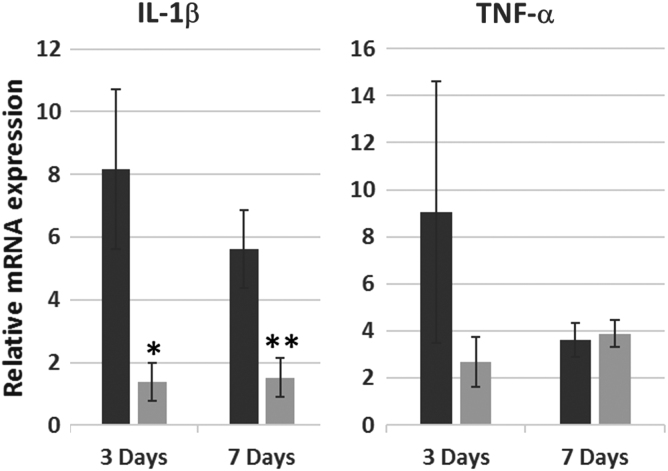
Relative IL-1β and TNF-α gene transcription after acute infarction and GeRP delivery of *Map4k4* siRNA. Relative abundance of *Il1b* and *Tnf* mRNA in infarcted hearts treated with injection of PBS (dark gray) versus GeRPs containing siRNA to *Map4k4* (light gray) at 3 and 7 days after infarction and intramyocardial injection. Values are expressed as mean fold changes (± SEM) relative to normal noninfarcted control hearts. *p = 0.06 versus PBS-treated infarcted hearts at 3 days; **p = 0.05 versus PBS-treated infarcted hearts at 7 days. IL, interleukin; mRNA, messenger RNA.

In comparison with infarcted, PBS-treated hearts, hearts injected with *Map4k4-*siRNA-loaded GeRPs had 73% lower levels of proinflammatory *IL-1β (Il1b)* transcripts at 3 days. A 69% reduction persisted out to 7 days (*p* = 0.05; Fig. 4). The postinfarct rise in TNF-α (*Tnf*) transcription at 3 days was also diminished by GeRP treatment (75% reduction; Fig. 4). Although differences in TNF-α (*Tnf*) transcription were lost by day 7 in whole heart specimens, evidence of persistent TNF-α suppression within the local infarct zone was observed on histology (Supplementary Fig. S3).

**FIG. 5.**
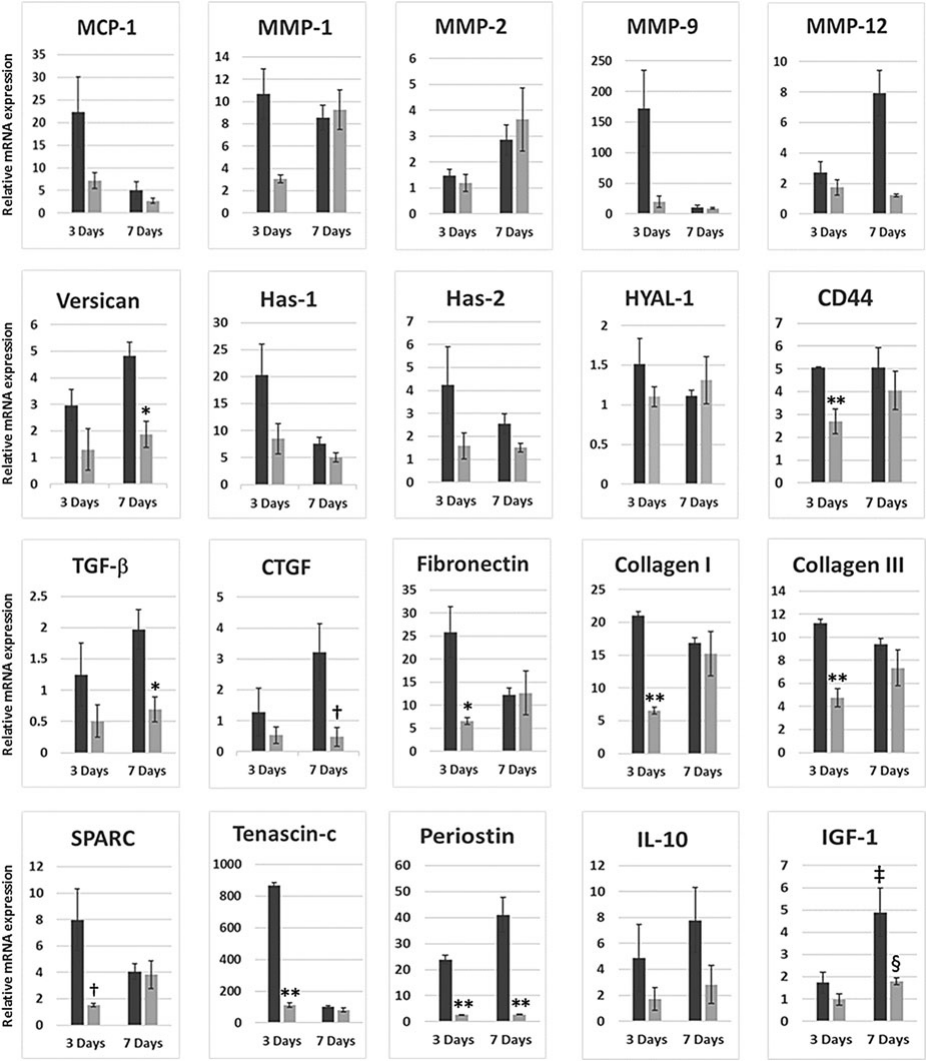
Relative gene transcription for factors related to infarct healing following acute infarction and GeRP delivery of *Map4k4* siRNA. Relative abundance of mRNA across whole infarcted hearts treated with injection of PBS (dark gray) versus GeRPs containing siRNA to *Map4k4* (light gray) at 3 and 7 days after acute infarction and intramyocardial injection. All values are expressed as mean fold changes (± SEM) relative to normal noninfarcted control hearts. *p < 0.05 versus PBS-treated infarcted hearts at the same time point; **p < 0.001 versus PBS-treated infarcted hearts at the same time point; ^†^*p* = 0.08 versus PBS-treated hearts on the given day; ^‡^*p* < 0.05 versus PBS-treated hearts at 3 days; ^§^*p* = 0.05 versus GeRP-treated hearts at 3 days.

Relative expression of MCP-1 (*Ccl2*) transcripts in infarcted hearts treated with *Map4k4*-siRNA-GeRPs was 68% lower at 3 days and 47% lower at 7 days than in PBS-treated infarcted hearts (Fig. 5).

### Impact of *Map4k4*-siRNA-loaded GeRP delivery on extracellular matrix remodeling

GeRP delivery into infarct zones affected key matrix and matricellular protein transcripts across the entire heart (Fig. 5).^14,15^ Among the MMPs tested, transcripts of *Mmp1* and *Mmp9* were both substantially reduced in GeRP-treated hearts by day 3, by 71% and 88%, respectively, versus PBS-treated, infarcted hearts. By day 7, levels of both were in line with infarcted controls. In GeRP-treated hearts, transcripts of *Mmp12* were 85% lower than in infarcted controls by day 7, effectively at noninfarcted baseline levels. *Mmp2* was not altered.

**FIG. 6.**
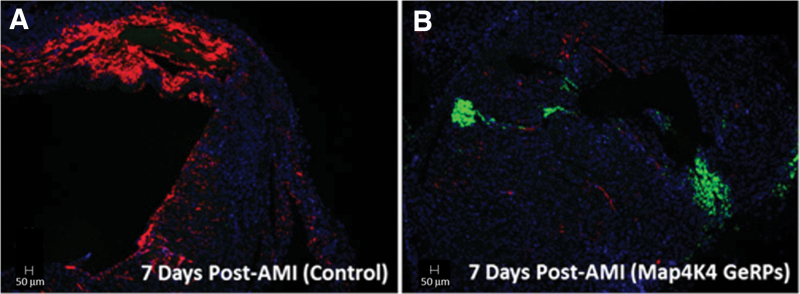
Myocardial HA content on histology at 7 days after acute infarction. Biotin-conjugated HA-binding protein (red) was used to visualize HA deposition in infarct areas in representative cross-sections of whole hearts at 7 days after infarction and GeRP or PBS injections. Extensive HA accumulation was found in the infarct area in PBS-treated hearts, consistent with an HA-rich (proinflammatory) provisional matrix **(A),** whereas minimal HA deposition was seen in infarcts treated with GeRPs containing *Map4k4* siRNA **(B)**. GeRP particles within the infarct area were identified by their incorporated fluorescein labels (green), as seen in the GeRP-treated heart **(B)**, but not present in the PBS-treated heart **(A)**. HA, hyaluronan.

The proteoglycan versican and its binding partner, HA, are primary components of the provisional matrix produced by infiltrating myeloid cells, both highly expressed and colocalized in infarct areas.^16–18^ Versican (*Vcan*) transcription in GeRP-treated hearts was reduced by 56% at 3 days, and by 61% at 7 days versus infarcted controls (*p* < 0.05). Effects on HA appeared to be mediated through early decreases in the HA synthases, *Has1* and *Has2*, whereas *Hyal1*, encoding the enzyme for HA degradation, was essentially unchanged. Transcription of cell-surface HA receptor gene, *Cd44*, was also halved at 3 days in GeRP-treated hearts (47% decrease, *p* = 0.001 vs. PBS-treated controls), which reversed by 7 days. The consequences of these transcriptional changes on extracellular matrix deposition were further evaluated by HA accumulation on histology in 7-day heart specimens. Abundant HA expression was seen in the provisional matrix within the infarct region in PBS-injected hearts, but was hardly detectable in infarcts injected with *Map4k4*-siRNA-loaded GeRPs (Fig. 6).

Profibrotic factors TGF-β1 and CTGF were both altered by GeRP-delivered *Map4k4* siRNA. TGF-β1 (*Tgfb1*) transcripts in hearts injected with *Map4k4*-siRNA-loaded GeRPs were reduced by 59% at 3 days, and by 65% at 7 days versus infarcted controls (*p* = 0.03, Fig. 5). *Ctgf* mRNA levels in GeRP-treated hearts were 58% lower at 3 days, and, by 7 days, CTGF expression was inhibited by 85% (*p* = 0.08). The postinfarct surge in fibronectin (*Fn1*) expression was also prevented by *Map4k4-*siRNA-GeRPs with a 75% lower level seen at 3 days (*p* = 0.04 vs. PBS-treated hearts). GeRP treatment additionally reduced expression of both collagen I (*Col1a1*) and III (*Col3a1*) genes at 3 days (by 69% [*p* < 0.0001] and 58% [*p* = 0.0002], respectively, vs. PBS controls). By day 7, fibronectin and collagens I and III expression levels were equivalent to those in infarcted PBS-treated hearts.

Postinfarct cell–matrix interactions are orchestrated by regulatory matricellular glycoproteins such as periostin, tenascin-c, and SPARC,^19^ all substantially diminished by GeRP treatment. Gene expression of the collagen-binding matricellular protein, SPARC, was reduced by 81% at 3 days (*p* = 0.08 vs. PBS-treated hearts), tenascin-c by 87% (*p* < 0.0001), and periostin by 89% (*p* < 0.0001). By day 7, SPARC (*Sparc*) and tenascin-c (*Tnc*) had been restored to standard postinfarct levels, whereas periostin (*Postn*) transcription remained significantly suppressed (89% reduction, *p* = 0.005 vs. PBS-treated infarcted hearts).

Between days 4 and 8 after AMI, late-appearing factors are also elaborated, here represented by IGF-1 and IL-10.^20–22^ IGF-1 (*Igf1*) expression levels increased markedly between days 3 and 7 in both PBS-treated and GeRP-treated infarcted hearts (each *p* ≤ 0.05; Fig. 5). A similar phenomenon was observed with IL-10 (*Il10*), but increases did not reach significance at this sample size. In GeRP-treated hearts, although *Igf1* and *Il10* transcripts increased by 85% and 65%, respectively, between days 3 and 7, levels remained lower than in PBS-treated infarcted hearts at day 7. Even so, these levels were still effectively doubled over those in noninfarcted controls.

### Intramyocardial injection of GeRPs was not associated with early ventricular rupture

No animals undergoing intramyocardial GeRP injections with *Map4k4*-directed siRNA had evidence of ventricular rupture by 7 days.

## Discussion

Previous trafficking studies have shown that few intracardiac macrophages exit the heart after AMI.^13^ Instead, they largely remain within the heart and die *in situ*.^13,23^ Here, similarly low rates of systemic dissemination were confirmed among the intracardiac macrophages that had phagocytosed infarct-injected GeRP particles. We capitalized on this inherent tendency for heart retention of differentiated macrophages to develop a method for *in situ* modification of the infarct microenvironment using infarct-injected GeRPs as a platform for siRNA delivery.

Other laboratories have utilized intravenous delivery of siRNA-containing nanoparticles to target postinfarct myocardial monocyte populations.^24–27^ After intravenous delivery, the largest populations of particle-containing cells were found in the spleen, liver, kidney, lymph nodes, and marrow, changing splenic monocyte expression and raising the potential for systemic immune effects. In comparison, direct intramyocardial particle delivery might provide a means to selectively modify the infarct microenvironment with less risk of off-target accumulation and consequences.

The 69–89% relative reductions in transcripts for Map4k4, IL-1β, and TNF-α achieved at 3 days after a single intramyocardial injection of siRNA-loaded GeRPs were consistent with those reported in peritoneal macrophages following a week of oral GeRP dosing.^4^ More profound inhibition of whole-heart *Map4k4* expression was not expected, given that (nontargeted) cardiomyocytes also produce Map4k4.^28^ Peak siRNA effects at 3 days, with persistence to 7 days in several instances (Map4k4, IL-1β, versican, TGF-β, CTGF, periostin), were in line with an expected half-life of 8 days for GeRP-delivered siRNA in tissues.^4^

Macrophage phenotypes across the entire heart were not markedly changed, although leukocyte populations within the infarct, i.e., at the site of GeRP delivery, were not specifically examined. Consistent with the goal of maintaining macrophage viability, phagocytosis of GeRP microparticles and/or *Map4k4* siRNA did not result in appreciable macrophage depletion. Rather, substantial functional alterations in the secretome were seen across the myocardium. Of note, a single dose of intramyocardial *Map4k4-*siRNA-loaded GeRPs reduced the transcription of a surprisingly broad spectrum of factors with relevance for infarct healing. MCP-1 and MMP-9 facilitate inflammatory cell recruitment from the circulation,^29–31^ whereas versican and HA mediate inflammatory cell trafficking through the infarct matrix.^16–19,32^ MMPs actuate matrix degradation,^33^ whereas TGF-β,^34^ CTGF,^35^ fibronectin,^36^ tenascin-c,^37^ SPARC,^38,39^ and periostin^40–42^ all contribute to myofibroblast activation, collagen deposition, and cardiac fibrosis. Together, diminished expression of this complementary combination of factors should predispose toward a less inflammatory provisional matrix, preservation of matrix structural integrity, and reduced risk for late fibrosis. Supporting this premise was the marked reduction in HA accumulation demonstrated in the provisional matrix at 7 days in GeRP-treated hearts (Fig. 6). However, definitive conclusions about the value of early *Map4k4* knockdown as a target in cardiac remodeling cannot be drawn from these limited pilots. Further experiments with longer durations of follow-up in larger groups will be essential to determine final effects on late cardiac matrix structure and ventricular function, as well as on macrophage phenotypes and cytokine elaboration within the specific infarct microenvironment.

It is noteworthy that no instances of cardiac rupture occurred by the 7-day time point. Earlier work has shown that direct inhibition of IL-1β,^43^ TNF-α,^44,45^ MMP-9,^31^ MMP-12,^46^ periostin,^47,48^ and SPARC^49^ can each lead to ventricular dilation and/or rupture within the first week postinfarct. The apparent relative safety of GeRP delivery might be ascribed to the short half-life of siRNA and to decremental reductions, rather than elimination, of inflammatory mediators and growth factors. In addition, structural factors, such as the collagens, recovered to expected transcription levels by 7 days. Although postinfarct rises in IGF-1 expression were dampened (or delayed) by GeRP treatment, they were still present, and the accompanying phenotypic switch from proinflammatory to reparative phenotypes in macrophages occurred unchecked.

Overall, these pilots demonstrate that macrophage *Map4k4* silencing appears a promising means to modulate the early infarct microenvironment after AMI. In clinical translation, postinfarct GeRP delivery could be accomplished with guided catheter-based intramyocardial injections^50^ performed at the time of coronary interventional procedures. More importantly, however, this platform could be utilized for any limited-duration RNAi strategy. If combined with cell transplantation for heart failure, or incorporated into engineered matrices, macrophage targets might be tailored toward survival and/or proliferation of implanted cells. In conclusion, these experiments demonstrate that intramyocardial GeRP injections may be a new and potentially clinically relevant means for localized and selective RNA delivery to intracardiac M/M populations.

## Supplementary Material

Supplemental data

Supplemental data

Supplemental data

## Abbreviations Used

AMI: acute myocardial infarction
cDNA: complementary DNA
CTGF: connective tissue growth factor
GeRPs: glucan-encapsulated small interfering RNA-containing particles
HA: hyaluronan
IGF-1: insulin-like growth factor-1
IL: interleukin
M/Ms: macrophages and monocytes
Map4k4: mitogen-activated protein kinase kinase kinase kinase 4
MCP-1: monocyte chemoattractant protein-1
MMP: matrix metalloproteinase
mRNA: messenger RNA
PBS: phosphate-buffered saline
PCR: polymerase chain reaction
PFA: paraformaldehyde
RNAi: RNA interference
rNRA: ribosomal RNA
SEM: standard error of the mean
siRNA: small interfering RNA
TGF-β: transforming growth factor beta
