# Supplementary material for: Immunotherapy for Infarcts: In Vivo Postinfarction Macrophage Modulation Using Intramyocardial Microparticle Delivery of Map4k4 Small Interfering RNA
Source: Biores Open Access. 2020 Dec 2;9(1):258–68. doi: 10.1089/biores.2020.0037 (PMC7757732; doi:10.1089/biores.2020.0037)
Supplement: Supplemental data [file Supp_Fig1.pdf]

SUPPLEMENTARY DATA

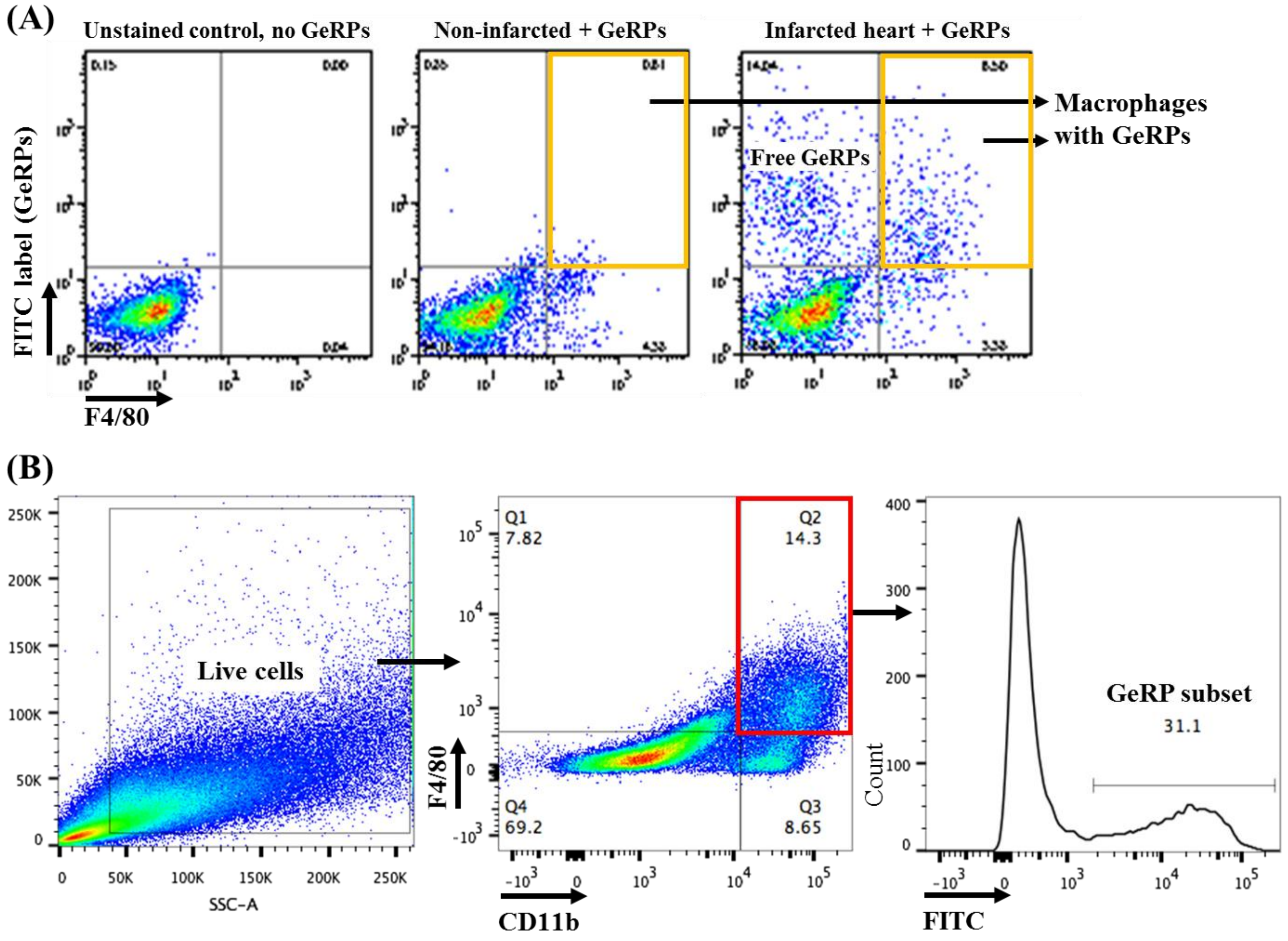

SUPPLEMENTARY FIG. S1. **(A)** Representative flow cytometry plots of whole heart isolates in experiments using empty GeRPs without siRNA. The left panel illustrates a control heart without infarction, GeRP injection, or F4/80 staining. The center panel shows a non-infarcted heart at 7 days following intramyocardial GeRP injection. The right panel exemplifies an infarcted heart at 7 days following infarction and intra-infarct GeRP injection. Orange outlines capture the F4/80^{+} cells co-localized with FITC fluorescence, indicative of intra-cardiac macrophages with ingested FITC-labeled GeRP particles. **(B)** Representative flow cytometry plot illustrating the method used to calculate the proportion of CD11b-gated F4/80^{+} cells containing FITC^{+} siRNA-loaded GeRPs in infarcted, GeRP-treated hearts. All live cardiac cells (left panel) were sorted into CD11b^{+}-gated F4/80^{+} macrophages (center) which were then assayed for FITC-containing GeRPs (right panel).
