# Supplementary material for: Immunotherapy for Infarcts: In Vivo Postinfarction Macrophage Modulation Using Intramyocardial Microparticle Delivery of Map4k4 Small Interfering RNA
Source: Biores Open Access. 2020 Dec 2;9(1):258–68. doi: 10.1089/biores.2020.0037 (PMC7757732; doi:10.1089/biores.2020.0037)
Supplement: Supplemental data [file Supp_Fig2.pdf]

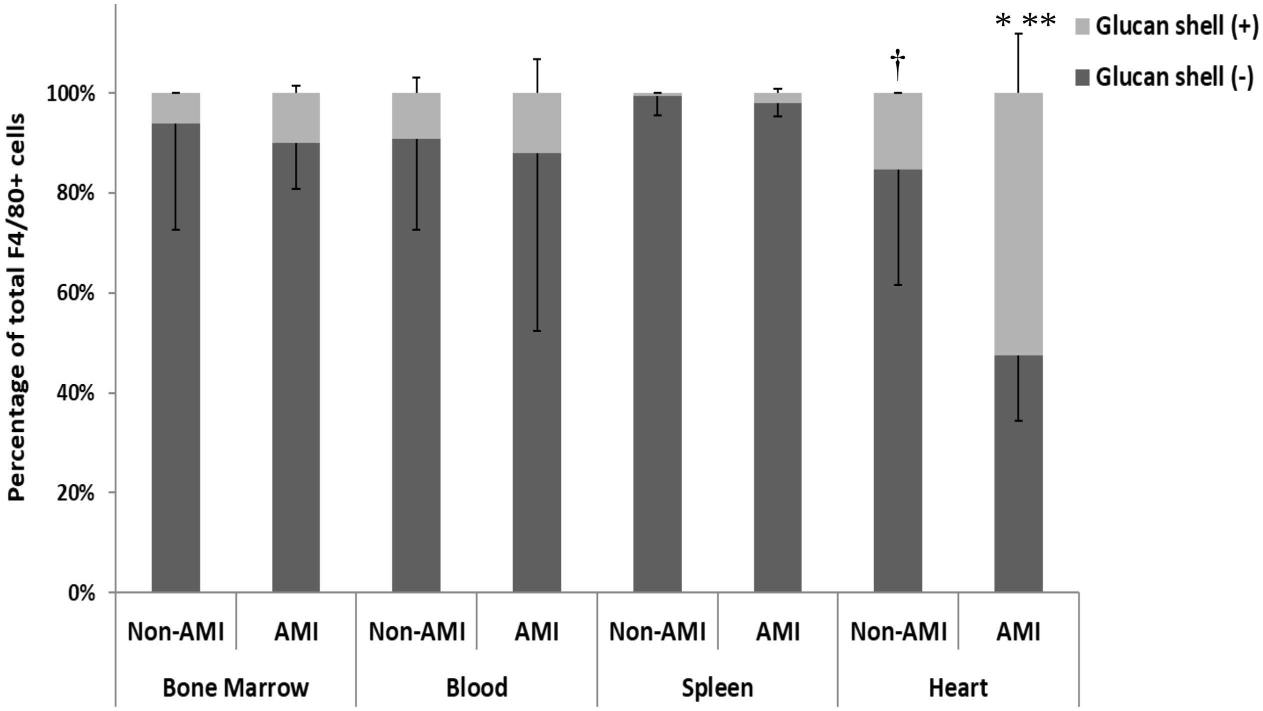

SUPPLEMENTARY FIG. S2. Percentage of total macrophages with ingested GeRPs in various organs. Empty GeRP particles were injected into hearts with acute myocardial infarcts (AMI) or into normal ventricles (non-AMI). CD11b$^+$/F4/80$^+$ macrophage populations in whole hearts, bone marrow, peripheral blood, and spleen were assessed by flow cytometry at 7 days following infarction and injection. Those with phagocytosed GeRPs were identified by the FITC-labeled β-glucan shell. Populations of GeRP-containing macrophages were significantly greater in infarcted hearts than in non-infarcted hearts, and greater than the small numbers of GeRP-containing macrophages that had trafficked to extracardiac sites. Even in the absence of infarction, GeRP-containing macrophages were significantly greater in hearts than in extracardiac sites, demonstrating low levels of dissemination of intra-cardiac macrophages after phagocytosis of GeRPs delivered into the myocardium.

* $p < 0.01$ infarcted hearts vs. non-infarcted hearts

**$p < 0.0001$ vs. all extracardiac sites in animals with infarcts

† $p < 0.03$ vs. all extracardiac sites in animals without infarcts
