# Supplementary material for: Immunotherapy for Infarcts: In Vivo Postinfarction Macrophage Modulation Using Intramyocardial Microparticle Delivery of Map4k4 Small Interfering RNA
Source: Biores Open Access. 2020 Dec 2;9(1):258–68. doi: 10.1089/biores.2020.0037 (PMC7757732; doi:10.1089/biores.2020.0037)
Supplement: Supplemental data [file Supp_Fig3.pdf]

SUPPLEMENTARY DATA

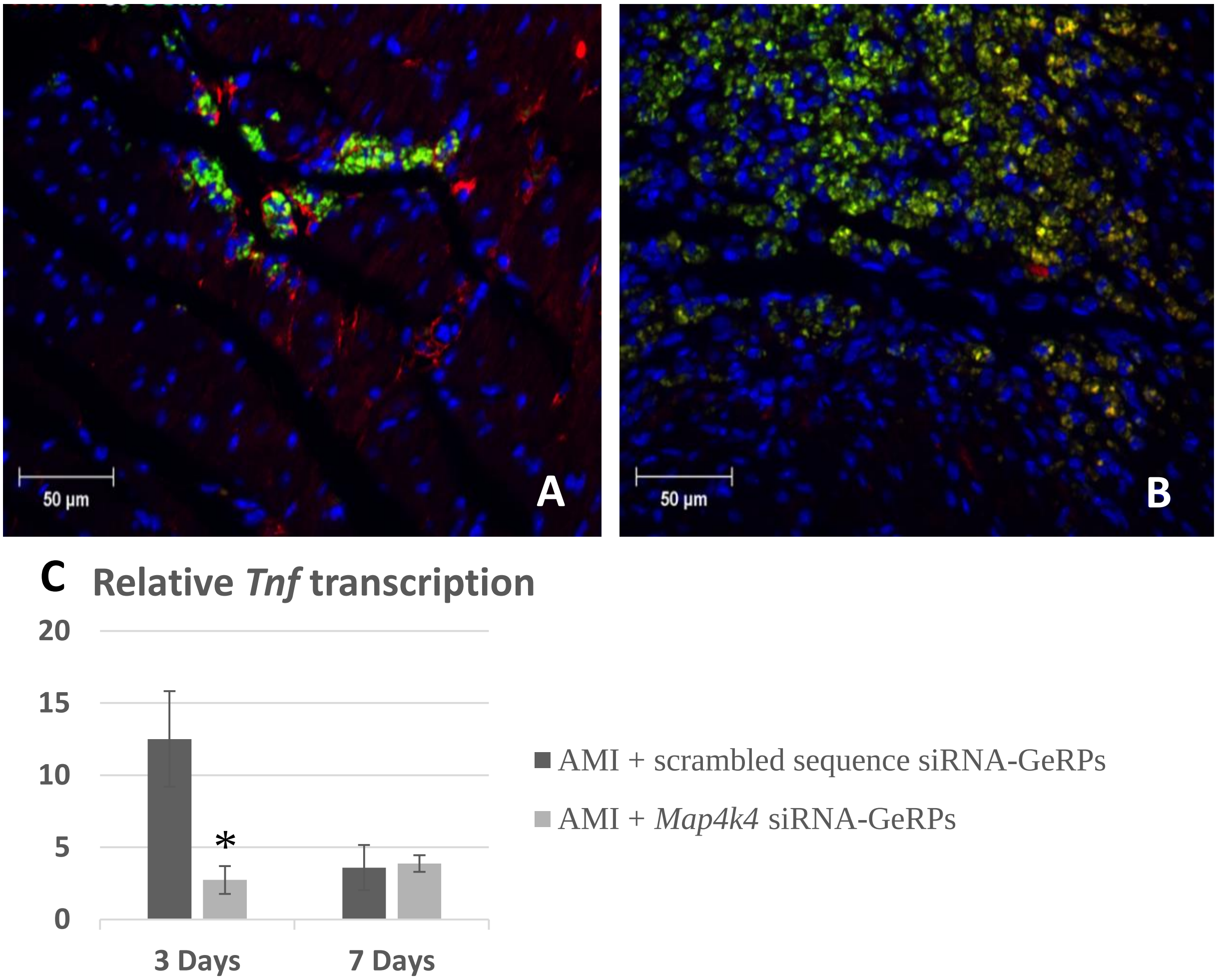

SUPPLEMENTARY FIG. S3. To clarify the TNF-α findings, another pilot was conducted, comparing intra-infarct delivery of *Map4k4*-targeted GeRPs to GeRPs containing scrambled siRNA (n=3), examining transcription and TNF-α protein expression in histologic cross-sections of whole hearts at 7 days following GeRP delivery. TNF-α was visualized in the infarct area by immunohistochemistry (red); GeRP particles were identified by their fluorescent label (green).

(A) Infarcted heart injected on Day 0 with GeRPs containing scrambled sequence siRNA.

(B) Infarcted heart injected on Day 0 with GeRPs containing *Map4k4* siRNA, showing relatively less TNF-α protein expression co-localized with the *Map4k4*-targeted GeRPs.

(C) Whole heart *Tnf* transcripts at 3 and 7 days in hearts injected with scrambled sequence siRNA followed the same pattern seen in PBS-treated infarcted hearts. However, hearts with *Map4k4*-targeted GeRPs had a 78% reduction in transcript levels at 3 days (*$p < 0.05$) which was no longer apparent at 7 days. Nonetheless, differences in protein expression were still evident on histology at 7 days within the infarct area at the site of GeRP delivery.
